# Characterization of genome-wide association study data reveals spatiotemporal heterogeneity of mental disorders

**DOI:** 10.1186/s12920-020-00832-8

**Published:** 2020-12-28

**Authors:** Yulin Dai, Timothy D. O’Brien, Guangsheng Pei, Zhongming Zhao, Peilin Jia

**Affiliations:** 1 Center for Precision Health, School of Biomedical Informatics, The University of Texas Health Science Center at Houston, 7000 Fannin St. Suite 820, Houston, TX 77030 USA; 2 Human Genetics Center, School of Public Health, The University of Texas Health Science Center at Houston, Houston, TX 77030 USA; 3 MD Anderson Cancer Center UTHealth Graduate School of Biomedical Sciences, Houston, TX 77030 USA; 4 Department of Biomedical Informatics, Vanderbilt University Medical Center, Nashville, TN 37203 USA

**Keywords:** Mental disorder, Genome-Wide Association Study, Protein-protein interaction, Prenatal, *CTNNB1*, *LNX1*, Apoptosis, Immune

## Abstract

**Background:**

Psychiatric disorders such as schizophrenia (SCZ), bipolar disorder (BIP), major depressive disorder (MDD), attention deficit-hyperactivity disorder (ADHD), and autism spectrum disorder (ASD) are often related to brain development. Both shared and unique biological and neurodevelopmental processes have been reported to be involved in these disorders.

**Methods:**

In this work, we developed an integrative analysis framework to seek for the sensitive spatiotemporal point during brain development underlying each disorder. Specifically, we first identified spatiotemporal gene co-expression modules for four brain regions three developmental stages (prenatal, birth to 11 years old, and older than 13 years), totaling 12 spatiotemporal sites. By integrating GWAS summary statistics and the spatiotemporal co-expression modules, we characterized the risk genes and their co-expression partners for five disorders.

**Results:**

We found that SCZ and BIP, ASD and ADHD tend to cluster with each other and keep a distance from other psychiatric disorders. At the gene level, we identified several genes that were shared among the most significant modules, such as *CTNNB1* and *LNX1*, and a hub gene, *ATF2,* in multiple modules. Moreover, we pinpointed two spatiotemporal points in the prenatal stage with active expression activities and highlighted one postnatal point for BIP. Further functional analysis of the disorder-related module highlighted the apoptotic signaling pathway for ASD and the immune-related and cell-cell adhesion function for SCZ, respectively.

**Conclusion:**

Our study demonstrated the dynamic changes of disorder-related genes at the network level, shedding light on the spatiotemporal regulation during brain development.

## Background

Mental disorders are leading causes of disability and comprise a substantial financial burden on the economy. It is estimated that one out of every four American adults suffers from a mental disorder in any given year [1]. Epidemiological evidence has revealed that the experiences in the prenatal and early childhood periods are related to later wellness [2]. This period is characterized by rapid and highly dynamic processes unfolding in space and time, which will have a lasting impact on health, learning, and behavior throughout one’s whole life [3]. Common psychiatric disorders, such as schizophrenia (SCZ), bipolar disorder (BIP), major depressive disorder (MDD), attention deficit hyperactivity disorder (ADHD), and autism spectrum disorder (ASD) have been proved to have high inheritability by twins or family studies [4–7]. Many genome-wide association studies (GWAS) have been conducted for mental disorders to reveal the common genetic risk loci in population [5, 7–9]. These GWA studies discovered hundreds of loci significantly associated with these disorders. However, interpretation and fine mapping of GWAS loci remain a major challenge in the post-GWAS era.

Previous studies have shown many spatiotemporal features of these five mental disorders. For instance, the major brain regions affected in SCZ included the prefrontal cortex, the basal ganglia, and the limbic system [10–12]. SCZ related genes tended to be highly expressed during prenatal development [13]. BIP was found to be related to the amygdala, hippocampus, and prefrontal cortex region [14, 15]. Both SCZ and BIP patients have gray matter reductions in paralimbic regions (anterior cingulate and insula), the function of which is emotional processing [15]. MDD patients have been observed to have significantly lower hippocampal volumes comparing the brain to the normal controls’ hippocampal volumes [1]. ADHD has a prevalence of 5.3% in childhood (younger than 18 years old) [16]. Two-thirds of patients with an ADHD diagnosis in childhood will continue to have impairing symptoms throughout their lives [17]. Subcortical structure volume especially the size of the amygdala, smaller volumes of caudate, cerebellum, and frontal and temporal gray matter have been associated with greater symptom severity [18–20]. Lastly, brain volume overgrowth was linked to ASD [21]. Patterns of gene expression distinguishing frontal and temporal cortex could be observed in the brains of autism patients [22].

With these lines of prior knowledge, we expect to bridge the molecular evidence to the features of each disorder. In the previous study conducted by Psychiatric Genomic Consortium (PGC) Cross-Disorder Group [7], the authors have identified four genetic variants shared in the five mental disorders. Inspired by this work, we aimed systematically characterize the spatiotemporal expression features of disorder-associated genes for five mental disorders utilizing the BrainSpan (Atlas of the developing human brain) expression data with the temporal and spatial transcriptome dynamic changes for more than 16 developing brain tissues aging from 4 post-conceptual weeks (pcw) (prenatal) to 60+ year old [23]. We aimed to pinpoint the shared and unique genetic factors of these five common psychiatric disorders in specific spatiotemporal points critical to brain development.

## Methods

### GWAS summary results for five psychiatric disorders

GWAS summary statistics were downloaded from Psychiatric Genomic Consortium (PGC) Cross-Disorder Group for each of the five disorders [7]. All patients were of European ancestry and were diagnosed as each primary disorder of interest according to the criteria from the DSM third edition revised or fourth edition. Specifically, there are 4788 trio cases, 4788 trio pseudocontrols, 161 cases, 526 controls for autism spectrum disorder (ASD); 1947 trio cases, 1947 trio pseudocontrols, 840 cases, and 688 controls in attention deficit-hyperactivity disorder (ADHD); 6990 cases and 4820 controls in bipolar disorder (BIP); 9227 cases and 7383 controls in major depressive disorder (MDD); and 9379 case and 7736 controls in schizophrenia (SCZ). All individuals are of European ancestry and are diagnosed with corresponding criteria. There are 1.2 million SNPs in total after imputation on CEU + TSI Hapmap Phase 3 reference and only those SNPs with imputation quality (INFO > 0.4) were used for further analysis.

### Gene-based *p*-values from VEGAS

We used liftOver to convert the GWAS SNPs from hg18 to hg19 [24]. The updated list of SNPs was used to calculate gene-based *p*-values using Versatile Gene-based Association Study (VEGAS) (version 2) [25]. VEGAS considers multiple SNPs mapped to a gene and calculates an empirical *p*-value to estimate the association after correcting for linkage disequilibrium (LD) structures. For each gene, we considered the SNPs mapped to the gene body or its 50 kb flanking region. We used the European population from the 1000 Genomes Project as the reference panel to estimate LD.

### Brain expression data

Spatiotemporal gene expression data were downloaded from the BrainSpan Atlas [23]. Following previous works (Table S5) [26], we split the samples into 12 categories based on their distinctive spatial and temporal features, ranging in four brain regions and three developmental periods. The regions are frontal cortex (FC), sensory motor regions (SM), sub-cortical regions (SC), and temporal-parietal cortex (TP). The stages are stage 1 (prenatal), stage 2 (after birth to 11 years old), and stage 3 (older than 13 years). We considered a gene that was expressed if its RPKM (Reads Per Kilobase of transcript per Million mapped reads) value was greater than one in at least one sample at each spatiotemporal point.

### PPI and CoPPI networks

We built the reference human protein-protein interaction (PPI) network by combining data from the Human Protein Reference Database [27] and the STRING database [28]. After removing self-interactions and isolated nodes, the final PPI network included 10,314 nodes (i.e., proteins) and 51,637 edges (i.e., interactions). A CoPPI is defined as an edge-weighted PPI network, in which each edge is weighted by the co-expression of the two nodes using the expression data generated in each specific spatiotemporal site. The absolute value of the Pearson Correlation Coefficient (PCC) was used to measure the co-expression level between a pair of nodes. We removed those edges involving unexpressed nodes from the network.

### Determination of co-expression modules

We modified the Dense Module Search (DMS) algorithm developed in our previous works [29–31]. Briefly, we defined a module score as the average edge weight, i.e., $Em=\frac{\sum {e}_{PCC}}{\#\text{edges}}$, where *e*_*PCC*_ indicated the absolute PCC value for each edge in the module. We started with edges whose *e*_*PCC*_ was ≥0.5 and expanded the module by always including the best edge connected to the current module, until no surrounding edge could improve the module score. With such a design, all the resultant modules had a module score > 0.5 and all their component edges had *e*_*PCC*_ ≥ 0.5.

### Determination of disorder-specific co-expression modules

Gene-based *p*-values from VEGAS were mapped to their respective genes in each significant co-expression module per spatiotemporal point. A module Z-score was calculated for each co-expression module for each disorder by $Zm=\frac{\sum Gw}{\sqrt{\# genes}}$, where *Gw* = Φ^−1^(1 − *p*_*g*_) is the gene-based score computed from the probit function of the VEGAS *p*-values [32]. The modules with a larger z-score indicate there are more genetic implications from the disorder in these modules. These module scores were then normalized by $Zn=\frac{Zm- mean(Zm)}{sd(Zm)}$. *Zn* was used for the following analysis.

### Functional enrichment analysis

We used R package DAVID to conduct functional enrichment analysis for gene ontology biological process [33]. Briefly, we conducted a Bonferroni correction to adjust the multiple-testing for the 1658 gene ontology biological process terms, five mental disorders, and 12 spatiotemporal points. Thus, the significant raw *p*-value threshold is (0.05/1658/5/12 ~ 5.0 × 10^− 7^). We further performed the same DAVID functional enrichment analysis for non-MHC modules genes. The MHC genes are defined as the 548 genes within the 8 M MHC high linkage disequilibrium (LD) region (chr6: 25500000–33,500,000) on hg19 reference. All the codes were performed on R version 3.5.2.

## Results

### Overview of design and results

**Fig. 1.**
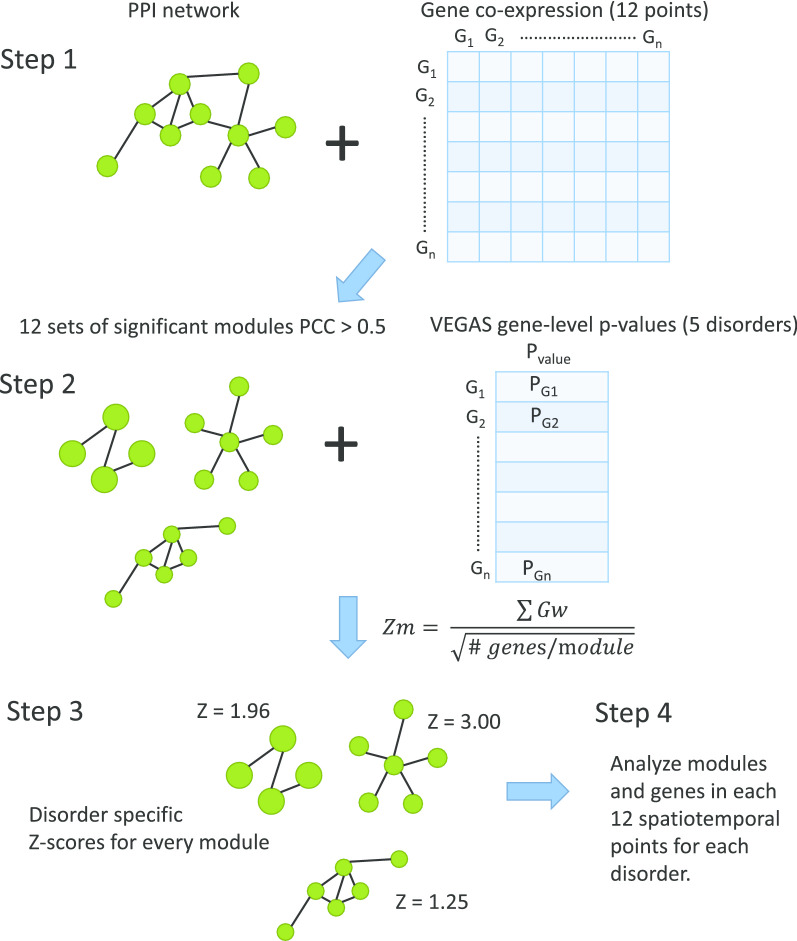
Working pipeline. Step1: construct reference Protein-Protein Interaction (PPI) network, overlay the co-expression on this PPI network and generate the co-expression PPI (COPPI) networks in 12 brain development spatiotemporal points. Step 2: integrate the five mental disorder Genome-Wide Association Study (GWAS) data with the co-expression modules. Step 3: calculate the disorder-specific z-score for each co-expression module. Step 4: comparison and functional analysis of the significant modules.

The outline of our work was illustrated in Fig. 1. Starting with a curated reference human PPI network, we overlaid gene co-expression relationships to each PPI pair for each temporal and spatial point, resulting in 12 CoPPI networks. We calculated PCC to measure the co-expression relationships among genes. The detailed classification and sample sizes of each spatiotemporal sites are presented in Table 1. We then constructed co-expression modules using our dense module searching (DMS) algorithm [29–31]. To identify highly co-expressed modules, we required all edges in a module to have an absolute PCC > 0.5 in the corresponding spatiotemporal site. We then overlaid gene-based z-scores calculated from GWAS summary statistics onto each spatiotemporal points and ranked modules according to the combined effect of co-expression and genetic associations. Notably, co-expression modules were identified for each spatiotemporal point, regardless of disorder data, and were comparable among disorders, whereas co-expression modules varied among spatiotemporal points.

**Table 1.** Number of significant co-expression modules (PCC > 0.5) per spatiotemporal point

Spatiotemporal point	Significant modules	Unique genes in modules
FC_ST1	1960	3822
FC_ST2	1527	3328
FC_ST3	1580	3485
SC_ST1	1869	3614
SC_ST2	1654	3551
SC_ST3	1775	3656
SM_ST1	1992	3806
SM_ST2	1533	3351
SM_ST3	1435	3193
TP_ST1	1844	3662
TP_ST2	1458	3256
TP_ST3	1416	3224

*ADHD* attention deficit hyperactivity disorder, *ASD* autism spectrum disorder, *BIP* bipolar disorder, *MDD* major depressive disorder, *SCZ* schizophrenia, *FC* frontal cortex region, *SM* sensory motor region, *SC* sub-cortical region, *TP* temporal-parietal cortex region, *ST1* stage 1 (prenatal), *ST2* stage 2 (after birth to 11 years old), *ST3* stage 3 (older than 13 years)

### Spatiotemporal co-expression modules in human brain development

The modules had an average of 4.22 nodes (range: 4–8) and 3.73 edges (range: 3–10). A total of 20,043 modules were identified for the 12 points. The SM region in the fetus had the most number (1992) of co-expression modules and the TP region in stage 3 had the least number (1416) of co-expression modules (Table 1). By comparing the module numbers in each brain region across different stages, we observed that ST1 had the highest number than the other two stages across different brain regions, indicating that ST1 was likely the most active stage in brain transcriptional activity.

### Identification of disorder-specific spatiotemporal modules

**Fig. 2.**
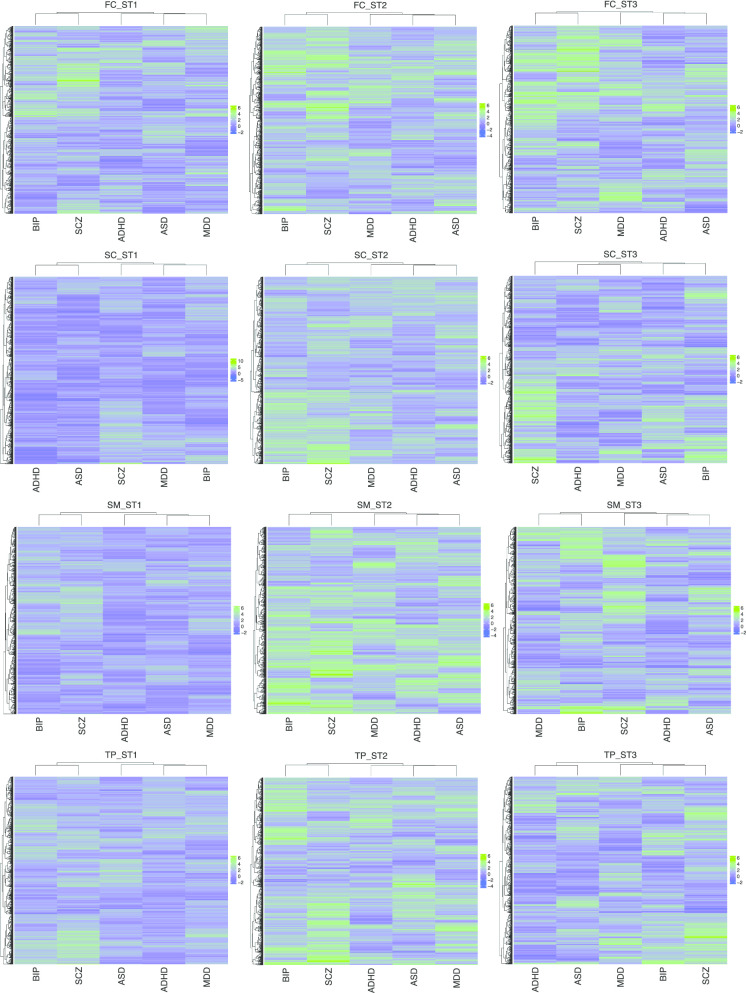
Comparison of temporal and spatial modules for five mental disorders. Heatmap based on the co-expression modules z-scores (module PCC > 0.5) in each spatiotemporal point for five mental disorders.

We next overlaid the gene-based z-scores (transformed from VEGAS *p*-values) onto the co-expression modules and ranked modules for each spatiotemporal site and each disorder. In this way, the module structure remained the same at each spatiotemporal point for all five disorders, whereas the modules were re-ordered according to their disorder associations. We explored the disorder correlations using the co-expression modules at each spatiotemporal point. Because the module list remained the same but only the module scores differed in each disorder, this analysis assessed the disorder correlation at the module level. As shown in Fig. 2, in 8 out of 12 points, SCZ and BIP formed a unique cluster distinct from the other three disorders, and in 10 out of 12 (83%) points, the two traits were clustered together. This remained true whether we used all modules or parts of the modules (e.g., the most 25% or the most 50% variable modules across all spatiotemporal sites) for the clustering analysis. This is consistent with previous studies that SCZ and BIP shared common polygenic variations [4, 14, 34–36]. More interestingly, we also observed ASD and ADHD formed in the same cluster away from the other three disorders in 7 out of 12 points and clustered together in 11 out of 12 (92%) points, indicating ASD and ADHD share more genetic background than the other three adult-onset disorders.

**Fig. 3.**
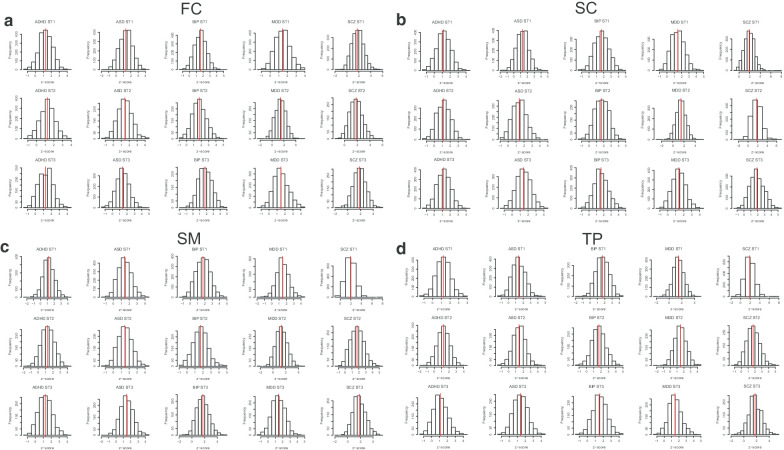
Comparison of the mean of module z scores for five mental disorders in four brain regions across three developmental stages. For **a**, **b**, **c**, **d**, each histogram represents the distribution of module z scores of one of each five mental disorders in each temporal point in FC, SC, SM, and TP brain regions, respectively.

Next, we checked the mean module z-score to explore the spatiotemporal points that have relatively high disorder effects from the GWAS signal (Fig. 3). We could observe ADHD ST2-SM have a higher mean z-score than the other two stages. ST2 in ASD shows a higher mean z-score than the other two stages in FC, SC, and TP, indicating the GWAS risk genes of ASD have a strong disturbance in ST2 (after birth to 11 years old). For BIP, ST3 (older than 13 years) was found to have higher z-scores in FC. ST3-FC has the highest z-score in MDD. ST1 (prenatal) in SCZ demonstrates the highest mean z-score in three brain developmental stages in SC, SM, and TP, suggesting SCZ GWAS risk genes have a strong effect in this stage across these three brain regions. Consistent findings could be observed by comparing the relative proportion of modules with Z_m_ > 1.96 to the total modules within 12 spatiotemporal points for each disorder (Additional file 1). We further normalized these module scores using standard normalization (see Methods). After normalization, we identified the number of modules in each disorder across all points with a module score (Z_n_) > 1.96 as significant modules and found that each disorder contained the significant modules ranging from 27 (96 unique genes in the modules) in ADHD (stage: ST2-SC) to 64 (196 genes) in ASD (ST1-SM) across all spatiotemporal points (Table 2).

**Table 2.** Summary of nominal significant disorder-associated co-expression modules (Zn > 1.96) and module genes

	# modules (# genes)				
	ADHD	ASD	BIP	MDD	SCZ
FC_ST1	46 (134)	50 (175)	47 (160)	**61** (**191**)	**58** (177)
FC_ST2	38 (141)	41 (142)	47 (137)	35 (138)	44 (145)
FC_ST3	42 (137)	50 (176)	45 (107)	51 (124)	41 (128)
SC_ST1	43 (131)	46 (145)	41 (114)	59 (166)	47 (158)
SC_ST2	27 (96)	52 (161)	39 (143)	38 (131)	47 (135)
SC_ST3	44 (143)	42 (126)	**58 (177)**	56 (177)	47 (145)
SM_ST1	**54 (185)**	**64 (196)**	53 (170)	59 **(**179**)**	55 (**195**)
SM_ST2	33 (113)	44 (147)	48 (162)	49 (144)	45 (154)
SM_ST3	46 (155)	48 (150)	49 (147)	35 (105)	49 (155)
TP_ST1	51 (155)	53 (151)	36 (121)	50 (147)	50 (152)
TP_ST2	34 (107)	45 (159)	44 (155)	34 (128)	45 (170)
TP_ST3	45 (128)	40 (140)	37 (136)	50 (146)	40 (139)

In Bold: The largest amount of co-expression modules or unique genes for each disorder in the 12 spatiotemporal points

*ADHD* attention deficit hyperactivity disorder, *ASD* autism spectrum disorder, *BIP* bipolar disorder, *MDD* major depressive disorder, *SCZ* schizophrenia, *FC* frontal cortex region, *SM* sensory motor region, *SC* sub-cortical region, *TP* temporal-parietal cortex region, *ST1* stage 1 (prenatal), *ST2* stage 2 (after birth to 11 years old), *ST3* stage 3 (older than 13 years)

### Weak overlap of modules and genes across the five disorders

We identified the overlap between the significantly identified modules across all points for the five disorders (Additional file 2A). We found that no modules overlapped between all five disorders at any spatiotemporal point. Only one module “*GRB2, LNX1, MAPK9, MUSTN1*” was found to be shared by four disorders (ASD, ADHD, SCZ, and BIP) in both ST3-SM and ST3-TP (Additional file 3, Table 3) [7]. Even though we observed a weak overlap in the specific modules across disorders, the genes contained in each module may overlap among the disorders. Therefore, we extracted all genes from all significant modules and determined their overlap in all points across all disorders (Table 4 & Additional file 2B). The most (196) and least (96) unique genes were extracted from ASD (ST1-SM) and ADHD (ST2-SC), respectively. We only observed two instances of a gene that was shared in all disorders. The first was for the ST2-SC (*CTNNB1*) and the second was for ST3-TP (*LNX1*), indicating these two genes might be involved in the pathogenesis of five mental disorders during these two spatiotemporal points. *CTNNB1* (Cadherin-Associated Protein Beta 1) has been proved to be related to abnormal brain development [37–40]. *LNX1* (Ligand of numb-protein X 1) is an E3 ubiquitin ligase for proteasomal degradation for NUMB protein, which is a key regulator of neurogenesis and neuronal differentiation [41]. Knockout *LNX1* and *LNX2* mice exhibited decreased anxiety-related behavior, though the mechanisms remained unknown [42]. The raw *p*-values of these two genes across five mental disorders were insignificant (p-value > 0.0001, Table 3), indicating that the risk genes in each disorder might have their mechanism influencing the co-expression of these two vital brain-development genes during certain spatiotemporal points. In sum, the results that most of the genes found in each disorder stayed unique to that disorder suggested unique genetic signatures for each disorder rather than shared.

**Table 3.** VEGAS gene-level p-values for key genes shared in five mental disorders

Gene\Disorder	ADHD	ASD	BIP	MDD	SCZ
*GRB2*	0.10	0.0017	0.021	0.30	4.65 × 10^−4^
*LNX1*	0.0063	0.012	0.0011	0.0045	0.0082
*MAPK9*	7.02 × 10^−4^	4.04 × 10^−4^	0.022	0.043	0.0046
*MUSTN1*	0.0062	4.72 × 10^− 4^	6.62 × 10^−6^	2.16 × 10^− 4^	3.80 × 10^−5^
*CTNNB1*	0.029	0.046	0.0081	8.08 × 10^− 4^	0.11
*ATF2*	0.0067	0.044	0.0014	0.0075	0.013

*ADHD* attention deficit hyperactivity disorder, *ASD* autism spectrum disorder, *BIP* bipolar disorder, *MDD* major depressive disorder, *SCZ* schizophrenia

**Table 4:** Hub genes identified from the top 10 significant modules (see Additional file 5)

Spatiotemporal point	ADHD	ASD	BIP	MDD	SCZ
FC_ST1	*ATF2*	*MYC*	*CAND1*	*E2F4*	*EP300*
FC_ST2	*STAT3*	*CUL3*	*CTNNB1*	*HTT*	*CREBBP*
FC_ST3	*ATF2*	*ACTB*	*APP*	*E2F4*	*EP300*
SC_ST1	*ATF2*	*MDM2*	*RBL2*	*TAF1*	*EP300*
SC_ST2	*ATF2*	*CUL3*	*ATF2*	*ATF2*	*EP300*
SC_ST3	*ATF2*	*CTNNB1*	*RBL2*	*DHX9*	*APP*
SM_ST1	*CTNNB1*	*RBL2*	*CUL2*	*NXF1*	*ATF2*
SM_ST2	*ATF2*	*CUL3*	*CCT4*	*UBE2I*	*MYC*
SM_ST3	*CTNNB1*	*CUL3*	*ATF2*	*ATF2*	*RBL2*
TP_ST1	*ATF2*	*ELAVL1*	*ATF2*	*XPO1*	*HIST1H4B*
TP_ST2	*BTRC*	*CUL3*	*ATF2*	*JUN*	*HIST1H4D*
TP_ST3	*ATF2*	*GRB2*	*CTNNB1*	*E2F4*	*GRB2*

*ADHD* attention deficit hyperactivity disorder, *ASD* autism spectrum disorder, *BIP* bipolar disorder, *MDD* major depressive disorder, *SCZ* schizophrenia, *FC* frontal cortex region, *SM* sensory motor region, *SC* sub-cortical region, *TP* temporal-parietal cortex region, *ST1* stage 1 (prenatal), *ST2* stage 2 (after birth to 11 years old), *ST3* stage 3 (older than 13 years)

### Functional annotation of significant modules

**Fig. 4.**
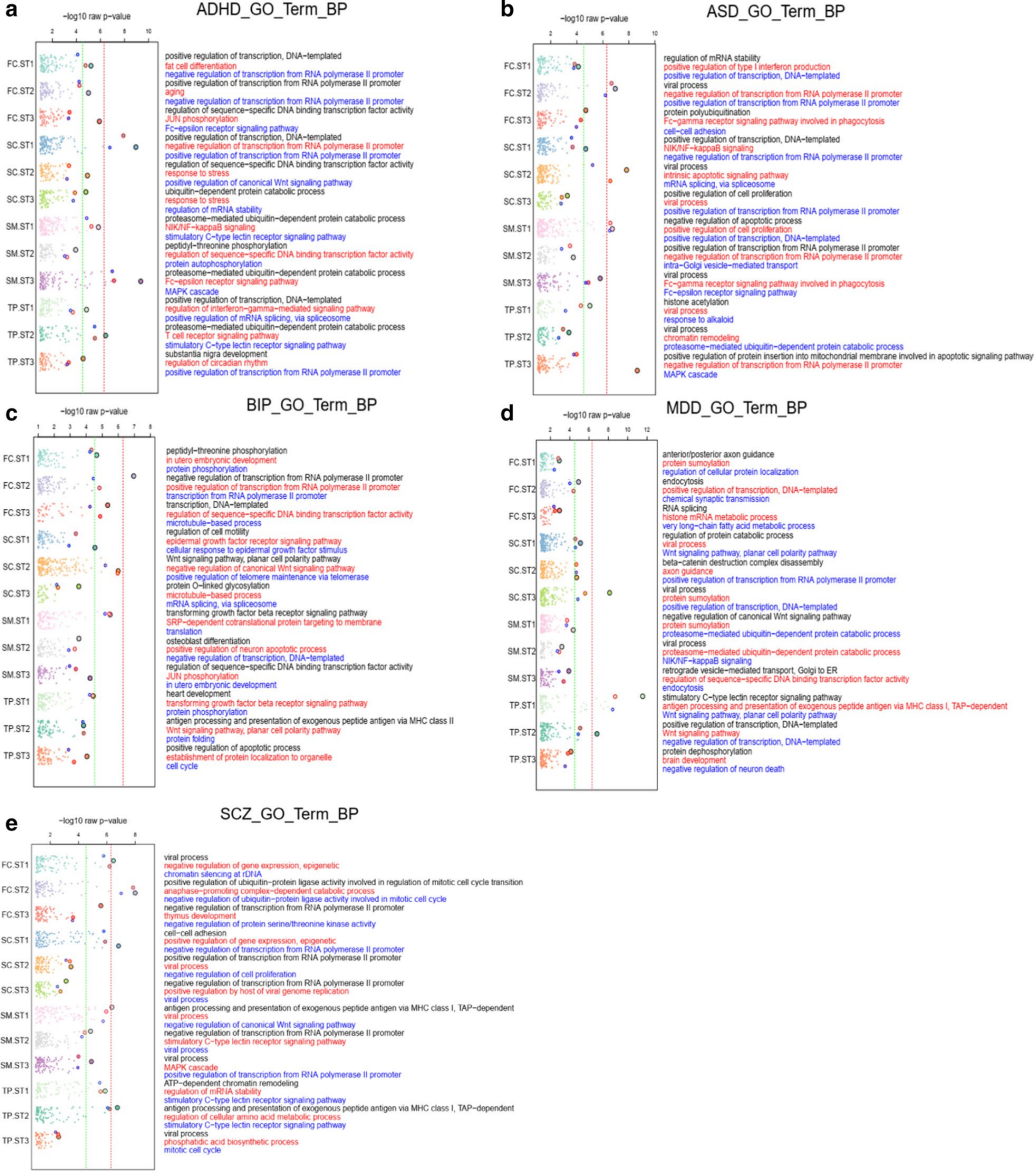
GO enrichment for genes in the top 10 significant modules 12 spatiotemporal points. Gene ontology term enrichment (biological process) analysis for five mental disorders in 12 spatiotemporal points. The top three GO terms were listed on the right for each spatiotemporal point in the order of “black”, “red”, and “blue”. Green dash indicated –log10 (*p*-value) after Bonferroni correction of all BP terms (1658). Red dash indicated significant –log10 (*p*-value) after Bonferroni correction of all BP terms and across 12 spatiotemporal points for 5 mental disorders. **a** ADHD: attention deficit hyperactivity disorder; **b** ASD: autism spectrum disorder; **c** BIP: bipolar disorder; **d** MDD: major depressive disorder; **e** SCZ: schizophrenia; FC: frontal cortex region, SM: sensory motor region, SC: sub-cortical region, TP: temporal-parietal cortex region, ST1: stage 1 (prenatal), ST2: stage 2 (after birth to 11 years old), ST3: stage 3 (older than 13 years).

To identify the biological roles of the genes in the significant modules, we performed functional enrichment analysis using DAVID (See Methods). We combined the genes from all significant modules for each point to find enriched pathways for each disorder illustrated in Fig. 4. In the enrichment study of GO terms for ADHD, we discovered genes were more likely to be enriched in proteasome-mediated ubiquitin-dependent protein catabolic process (GO:0043161) in the ST3-SM (p_raw_ = 4.0 × 10^− 10^) and ST2-TP (p_raw_ = 3.3 × 10^− 7^) regions. Regulation of transcription related functions were also found in ST1-SC (positive regulation of transcription, DNA-templated GO:0045893 p_raw_ = 1.0 × 10^− 9^). (Fig. 4a). For ASD, we discovered 3 significant terms in the ST1-SM (GO:0043066 negative regulation of apoptotic process p_raw_ = 1.7 × 10^− 7^, GO:0008284 positive regulation of cell proliferation p_raw_ = 2.5 × 10^− 7^) and ST2-SC (GO:0097193 intrinsic apoptotic signaling pathway p_raw_ = 2.6 × 10^− 7^) in early brain development, which were related to apoptotic and cell proliferation [21] (Fig. 4b). For BIP, the top significant terms in ST2-FC and ST2-SC were negative regulation of transcription from RNA polymerase II promoter (GO:0000122 p_raw_ = 1.0 × 10^− 7^) and Wnt signaling pathway, planar cell polarity pathway (GO:0060071 p_raw_ = 1.0 × 10^− 6^), respectively (Fig. 4c). MDD module genes were found enriched in ST3-SC and ST1-TP for immune-related pathways (viral process GO:0016032 p_raw_ = 1.0 × 10^− 8^, stimulatory C-type lectin receptor signaling pathway GO:0002223 p_raw_ = 2.5 × 10^− 12^, and antigen processing and presentation of exogenous peptide antigen via MHC class I, TAP-dependent GO:0002479 p_raw_ = 1.8 × 10^− 9^), suggesting the immune disturbance in brain TP and SC region could be the underlying etiology of MDD (Fig. 4d). Last but not the least, for SCZ, diverse Bonferroni-correction significant terms were found, e.g., the positive/negative ubiquitin-protein ligase activity in regulation of mitotic cell cycle in ST2-FC (GO:0051437 p_raw_ = 1.0 × 10^− 8^ and GO:0051436 p_raw_ = 1.0 × 10^− 7^); antigen processing and presentation of exogenous peptide antigen via MHC class I, TAP-dependent (GO:0002479) for ST1-SM (p_raw_ = 4.0 × 10^− 7^) and ST2-TP (p_raw_ = 1.7 × 10^− 7^), respectively; viral process (GO:0016032 p_raw_ = 3.2 × 10^− 7^) in ST1-FC, and cell-cell adhesion (GO:0007155 p_raw_ = 1.6 × 10^− 7^) in ST1-SC (Fig. 4e).

## Discussion

The determination of the biological basis for psychiatric disorders is important in terms of patient intervention and the potential basis for treatment options. In this study, we used a network approach to identify genes and their biological mechanisms underlying five psychiatric disorders: ADHD, ASD, BIP, MDD, and SCZ. By taking advantage of the comprehensive BrainSpan data with temporal and spatial gene expression profiles, we identified significant co-expression modules and interrogated their potential functions. Specifically, we pinpointed several spatiotemporal points that genetic disturbance of gene interaction networks might increase the risk of the onset of each psychiatric disorder during brain development. Our observations also suggested that the majority of genetic predisposition to these disorders was unique to each disorder, although shared genes were identified as well.

We found that SCZ and BIP were closely clustered in 10 out of the 12 investigated spatiotemporal sites, while ASD appeared to be distantly related to the other four disorders. We identified that neurodevelopmental ST1 has the most co-expression modules than other stages across four main brain regions, indicating the prenatal stage is the ‘busiest’ stage during brain development. Surprisingly, we only observed one nominal significant module composed of four genes “*GRB2, LNX1, MAPK9, MUSTN1*” shared by four disorders (ASD, ADHD, SCZ, and BIP) in both ST3-SM and ST3-TP. The gene *MUSTN1* has the most significant gene-level *p*-value (Table 3), which is nearby the genome-wide significant signal (rs2535629) from the previous meta-analysis of these five disorders [7]. A limited number of modules were shared in multiple disorders in each site, implying a much more complicated relationship among these disorders at the pathway/network level. At the gene level, we identified several genes that were shared among the most significant modules. Example genes included *CTNNB1*, a Wnt signaling gene; *LNX1*, an E3 ubiquitin-protein ligase; and a transcriptional factor *ATF2*. Genes with both strong associations and moderate/weak associations were found to interact with each other and form modules that led to the development of disorders.

**Fig. 5.**
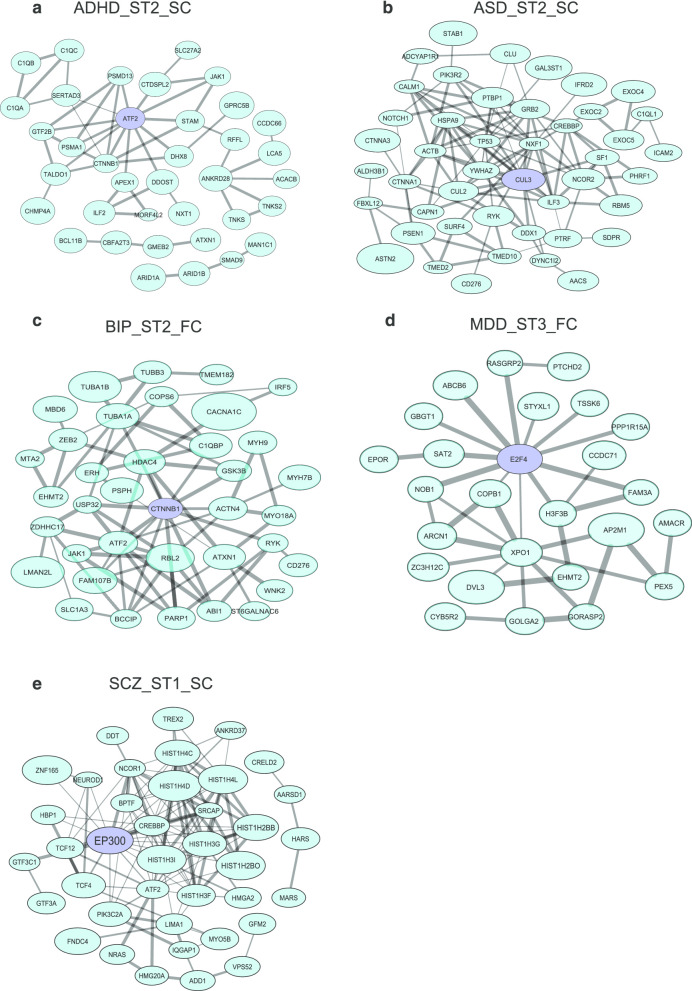
Representative subnetworks merged from the top 10 modules that were most significantly associated with each disorder. Subnetworks merged from the top 10 disorder-related significant modules. Nodes in purple indicated hub genes; nodes size is proportional to the corresponding GWAS gene p-value; edge width is proportional to the corresponding Pearson Correlation Coefficient of two genes. **a** ADHD_ST2_SC: attention deficit hyperactivity disorder stage 2 sub-cortical region; **b** ASD_ST2_SC: autism spectrum disorder stage 2 sub-cortical region; **c** BIP_ST2_FC: bipolar disorder stage 2 frontal cortex region; **d** MDD_ST3_FC: major depressive disorder stage 3 frontal cortex region; **e** SCZ_ST1_SC: schizophrenia stage 1 sub-cortical region. FC: frontal cortex region, SM: sensory motor region, SC: sub-cortical region, TP: temporal-parietal cortex region, ST1: stage 1 (prenatal), ST2: stage 2 (after birth to 11 years old), ST3: stage 3 (older than 13 years).

*CTNNB1* is a fundamental component of the canonical Wnt signaling pathways and controls cell growth and cell adhesion [43, 44]. Dysregulation of *CTNNB1* leads to abnormal brain development and defective dendritic morphogenesis [37–40]. Mutation in *CTNNB1* could to neurodevelopmental disorder [45]. In our results, *CTNNB1* was found in significant co-expression modules in all five disorders at ST2 in the SC region of the brain. Raw *p*-values of *CTNNB1* were not significant in VEGAS results (ADHD:0.029; ASD:0.046; BIP:0.0081, MDD:0.00081; SCZ: 0.11). *CTNNB1* was also found to be the hub node in several spatiotemporal points of ADHD, ASD, and BIP (Table 4 and Fig. 5), suggesting that it might play important roles in these spatiotemporal points of development. More interestingly, *CTNNB1* was found to be coexpressed with *HDAC4* and *CACNA1C* in the top modules in BIP ST2-FC spatiotemporal point (Fig. 5c). While the gene *CACNA1C* is also the genome-wide significant loci (rs1024582) shared among these five major psychiatric disorders in previous PGC cross-disorder work [7]. The gene *LNX1* was found in the top modules in the TP region ST3 (Table 4). *LNX1* was an insignificant gene based on the GWAS results (ADHD:0.0063; ASD:0.012; BIP:0.0011, MDD:0.0045; SCZ: 0.0082) (Table 3). *LNX1* was found to be involved in regulating the protein NUMB, which determines cell fates during development. Also, LNX1 was found to have interactions with presynaptic proteins ERC1, ERC2, and LIPRIN-αs (PPFIA1, PPFIA3), as well as the F-BAR domain proteins FCHSD2 (nervous wreck homolog) and SRGAP2 [42]. *ATF2* was found to be the hub node in 15 out of 60 disorder spatiotemporal points in the top 10 significant modules (Tables 3 and 4). This gene was a transcriptional activator that regulates the transcription of various genes involved in anti-apoptosis, cell growth, and DNA damage response. According to the gene expression during development in SZGR2 database [46], *ATF2* has higher expression before born than after born in the brain region, suggesting this gene was involved in regulating the fetus stage of brain development. None of the three genes (*LNX1*, *CTNNB1*, and *ATF2*) was significantly based on the GWAS results of the five disorders (Table 3). They were discovered by our approach mainly because these genes interact with other genes and jointly formed significant modules.

As shown in Table 2, all five mental disorders but BIP were found to have the largest amount of module Z_n_ in Stage 1 (prenatal stage). Interestingly, recent studies also revealed that psychiatric disorders relevant genes tend to be highly expressed in prenatal than postnatal stages [47]. Consistent with the number of significant co-expression modules in Table 1, we found that ST1-FC and ST1-SM tend to have the largest numbers of disorder-related modules, suggesting these two spatiotemporal points are the most curial stages and brain regions underlying these five mental disorders. Overlapping with our functional enrichment analysis result (Fig. 4), we highlighted negative regulation of the apoptotic process (GO:0043066), positive regulation of cell proliferation (GO:0008284) and positive regulation of transcription, DNA-templated (GO:0045893) in ST1-SM for ASD. These findings were aligned with the programmed cell death during neural development, suggesting spatiotemporal, quantitative errors raised by internal or external stimuli would lead to an abnormal number of neurons and pathological neural connections [21, 48, 49]. The viral process (GO:0016032) in ST1-FC and antigen process and presentation of exogenous peptide antigen via MHC class I, TAP-dependent (GO:0002479) in ST1-SM for SCZ. Recently, SCZ has been correlated to the dysregulation in prenatal brain development and immune response function [50–52].

Although we failed to identify any significantly enriched functions for ADHD, BIP, MDD in these two spatiotemporal points (ST1-FC and ST1-SM), we still observed several significant terms in other spatiotemporal points strongly supported by many known observations and studies. ADHD is featured with volume changes of subcortical structure, especially the size of amygdala, smaller volumes of caudate, cerebellum, and frontal and temporal gray matter. We identified the ubiquitin process ADHD is related to in ST3-SM and ST2-TP. Previous studies have shown BIP was associated with the abnormalities in the SC and FC regions [14, 15]. We identified two top terms, negative regulation of transcription from RNA polymerase II promoter and Wnt signaling pathway in ST2-FC and ST2-SC, respectively (Fig. 4c). MDD has high comorbidity (20–55%) with mesial temporal lobe epilepsy (MTLE) [53], which is associated with TP and SC regions [26]. Strikingly, multiple immune-related terms are highly enriched in these two regions among three stages (Fig. 4d), indicating that immune disruption in these regions during brain developments might lead to MDD and its co-occurring disorders.

Due to the complex LD structure in the major histocompatibility complex (MHC) region, we also conducted a supplementary analysis for those top modules excluding those MHC genes (Methods, Additional file 4). Briefly, we found most immune-related functions are not in the top three GOBP terms, except for antigen processing and presentation functions in BIP ST2-TP point, indicating BIP might be related to an immune-associated mechanism outside the MHC region. Interestingly, positive regulation of neuron death (GO:1901216 p_raw_ = 6.0 × 10^− 6^) was highlighted in the key point ST2-FC for BIP.

Lastly, some of the psychiatric disorders could be differentiated by their symptom patterns and course of illness, e.g. SCZ, BIP, and MDD. However, due to the stage and degree of disorder and shared underlying genetic risk factors, it is difficult to define a clear boundary for phenotyping the psychiatric disorders, such as ASD and ADHD [7], which also leads to the different statistical powers for different psychiatric GWAS and eventually hinders our comparison across disorders. Thus, we designed to explore the top 10 genetically impacted modules for each disorder in each spatiotemporal point. However, we also provided lists of genes for each disorder in each of the 12 spatiotemporal points with significant module z-score after multiple-test correction (Additional file 1).

## Conclusion

In this work, we developed a network-based module approach to investigate the cumulative impact of disorder-associated genes in different brain developmental stages across different brain regions. We pinpointed two known genetic risk factors (rs2535629 and rs1024582) in our spatiotemporal co-expression network and highlighted several hub genes, e.g., *CTNNB1* and *LNX1*, which likely played crucial regulatory roles in these disorders. Our results recapitalized the dynamic correlations among the five mental disorders and highlighted brain regions and developmental stages underlying disorder co-expressed modules and genes. For instance, the genes from ASD and SCZ modules are significantly enriched in the apoptotic signaling pathway in ST1-SM;immune-related and cell-cell adhesion function for SCZ are enriched in ST1-FC/SM and ST1-SC, respectively. Overall, our investigation of the developmental brain provides new understandings underlying the etiology of these five mental disorders.

## Supplementary Information

**Additional file 1.** Bubble diagram for the proportion of the modules Z_m_ > 1.96 in 12 spatiotemporal points. (A)(B)(C)(D)(E). Bubble diagram describing the proportion of the modules with Z_m_ > 1.96 to the total modules within 12 spatiotemporal points for every 5 mental disorders, respectively. The bubble size represents the relative proportion of 12 spatiotemporal points of each disorder.

**Additional file 2.** Venn diagram for genes from modules. (**A**) Venn diagram describing the overlapping modules of 12 spatiotemporal points (**B**) Venn diagram describing the overlapping genes from modules of 12 spatiotemporal points. Plots were generated by the online tool http://bioinformatics.psb.ugent.be/webtools/Venn/.

**Additional file 3.** Gene lists merged from the statistically significant modules. Each sheet contains the gene list corresponding to the five psychiatric diseases. (A) ADHD: attention deficit hyperactivity disorder; (B) ASD: autism spectrum disorder; (C) BIP: bipolar disorder; (D) MDD: major depressive disorder; (E) SCZ: schizophrenia; FC: frontal cortex region, SM: sensory motor region, SC: sub-cortical region, TP: temporal-parietal cortex region, ST1: stage 1 (prenatal), ST2: stage 2 (after birth to 11 years old), ST3: stage 3 (older than 13 years)

**Additional file 4.** GO enrichment for genes (non MHC genes) in the top 10 significant modules. Gene ontology term enrichment (biological process) analysis for five mental disorders in 12 spatiotemporal points. The top three GO terms were listed on the right for each spatiotemporal point in the order of “black”, “red”, and “blue”. Green dash indicated –log10 (*p*-value) after Bonferroni correction of all BP terms (2740). (A) ADHD: attention deficit hyperactivity disorder; (B) ASD: autism spectrum disorder; (C) BIP: bipolar disorder; (D) MDD: major depressive disorder; (E) SCZ: schizophrenia; FC: frontal cortex region, SM: sensory motor region, SC: sub-cortical region, TP: temporal-parietal cortex region, ST1: stage 1 (prenatal), ST2: stage 2 (after birth to 11 years old), ST3: stage 3 (older than 13 years)

**Additional file 5.** Edges weights for the top 10 modules for five psychiatric diseases in 12 spatiotemporal points. The first two columns are gene symbols, the third column is the Pearson Correlation Coefficient is the edge weight, and the last column is the corresponding Disorder spatiotemporal point. (A) ADHD: attention deficit hyperactivity disorder; (B) ASD: autism spectrum disorder; (C) BIP: bipolar disorder; (D) MDD: major depressive disorder; (E) SCZ: schizophrenia; FC: frontal cortex region, SM: sensory motor region, SC: sub-cortical region, TP: temporal-parietal cortex region, ST1: stage 1 (prenatal), ST2: stage 2 (after birth to 11 years old), ST3: stage 3 (older than 13 years)

## Data Availability

The Psychiatric Genomics Consortium Cross-Disorder Group data is available through the request from their website (https://www.med.unc.edu/pgc/pgc-workgroups/cross-disorder-group/). The BrainSpan Atlas expression profiles are available from their website (https://www.brainspan.org/static/download.html). All the datasets used and/or analyzed during the current study are available from the resources described in the Methods part.

## Abbreviations

ADHD: Attention deficit hyperactivity disorder
ASD: Autism spectrum disorder
BIP: Bipolar disorder
MDD: Major depressive disorder
SCZ: Schizophrenia
FC: Frontal cortex region
SM: Sensory motor region
SC: Sub-cortical region
TP: Temporal-parietal cortex region
ST1: Stage 1
ST2: Stage 2
ST3: Stage 3
GWAS: Genome-wide association studies
PGC: Psychiatric Genomic Consortium
pcw: Post-conceptual weeks
VEGAS: Versatile Gene-based Association Study
LD: Linkage disequilibrium
PPI: Protein-protein interaction
DMS: Dense module search
