# Supplementary figures and images for: Characterization of genome-wide association study data reveals spatiotemporal heterogeneity of mental disorders
Source: BMC Med Genomics. 2020 Dec 28;13(Suppl 11):192. doi: 10.1186/s12920-020-00832-8 (PMC7771094; doi:10.1186/s12920-020-00832-8)

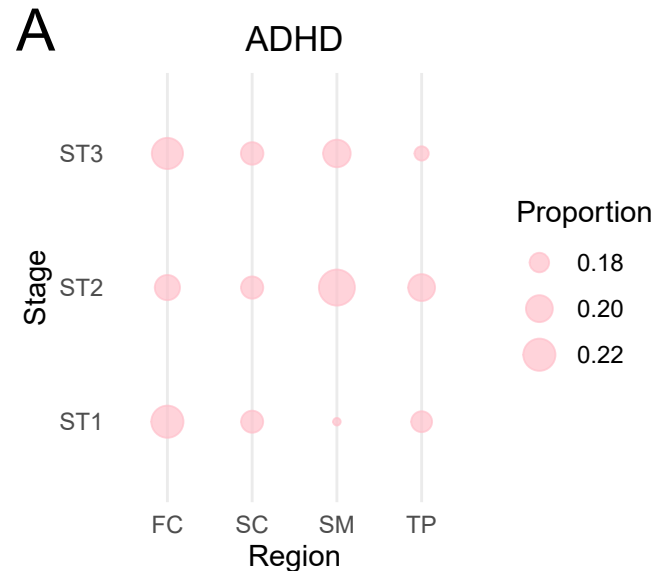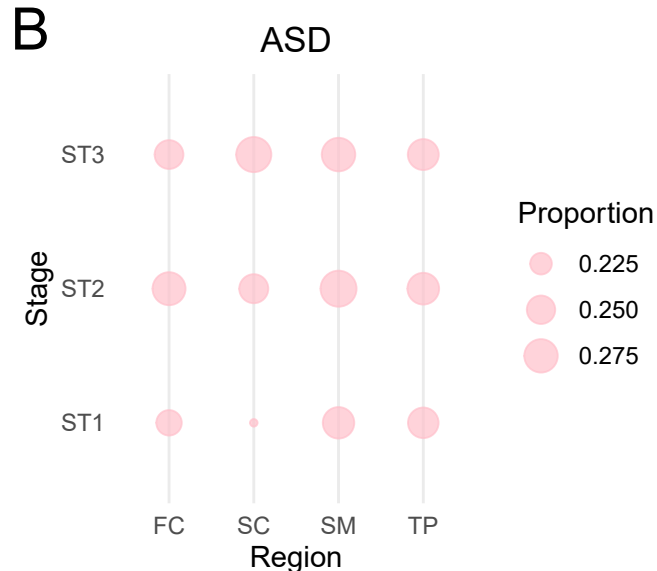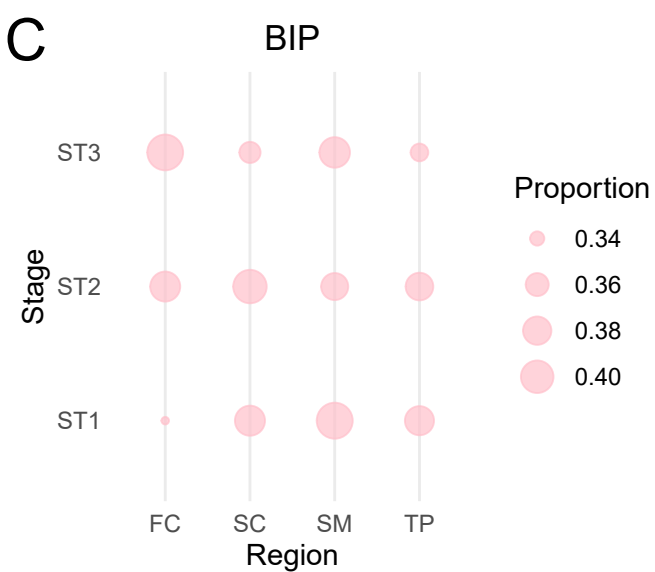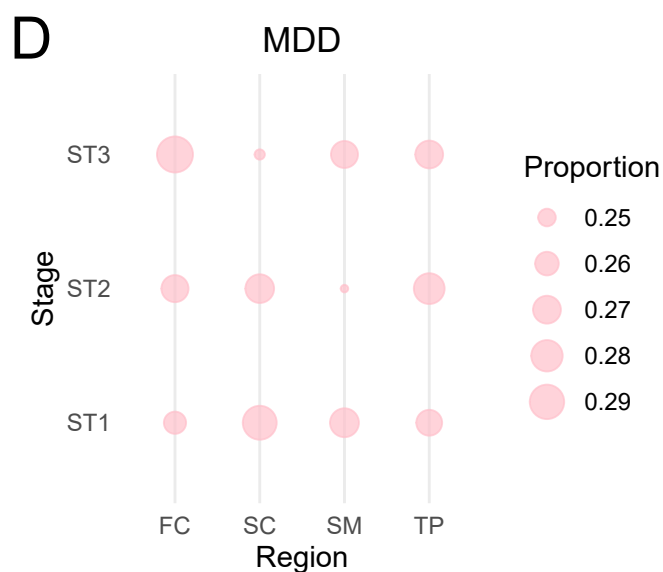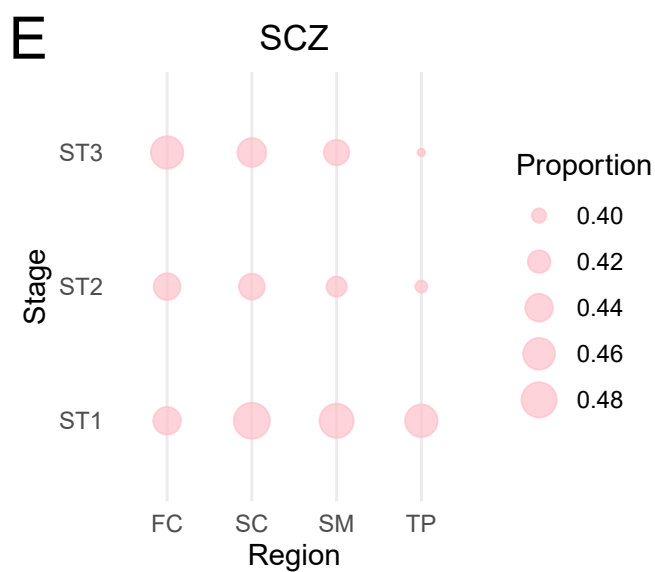

Supplement: Supplementary file 1 — Additional file 1. Bubble diagram for the proportion of the modules Zm > 1.96 in 12 spatiotemporal points. (A)(B)(C)(D)(E). Bubble diagram describing the proportion of the modules with Zm > 1.96 to the total modules within 12 spatiotemporal points for every 5 mental disorders, respectively. The bubble size represents the relative proportion of 12 spatiotemporal points of each disorder. [file 12920_2020_832_MOESM1_ESM.pdf]

A

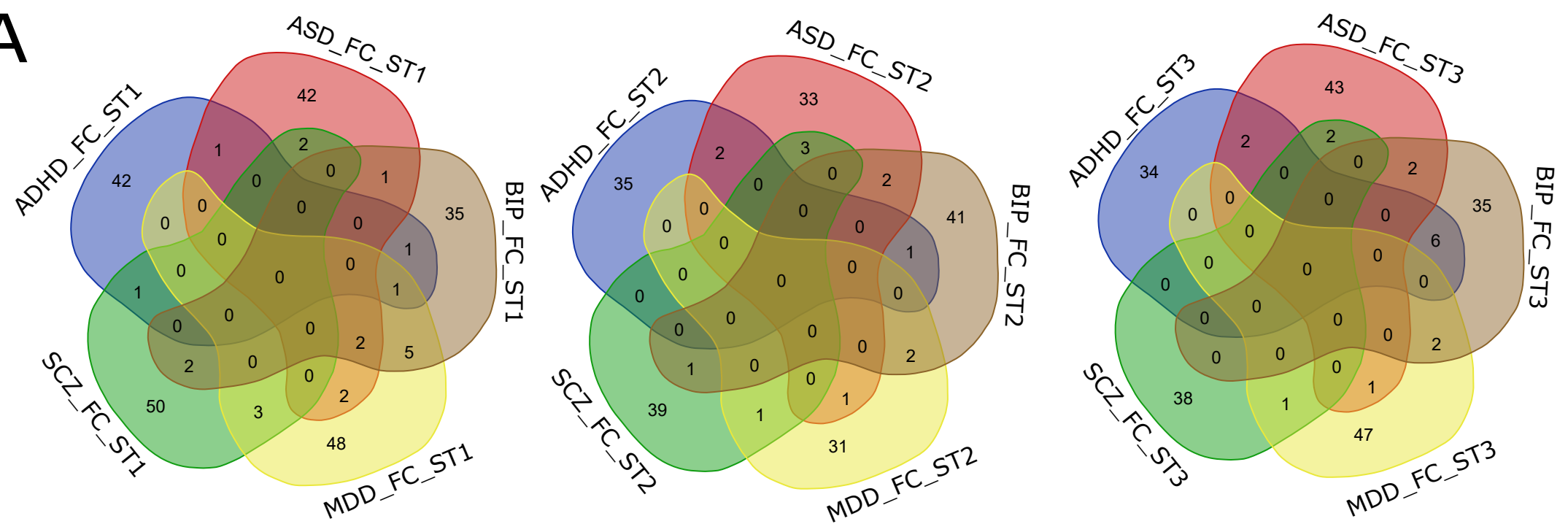

B

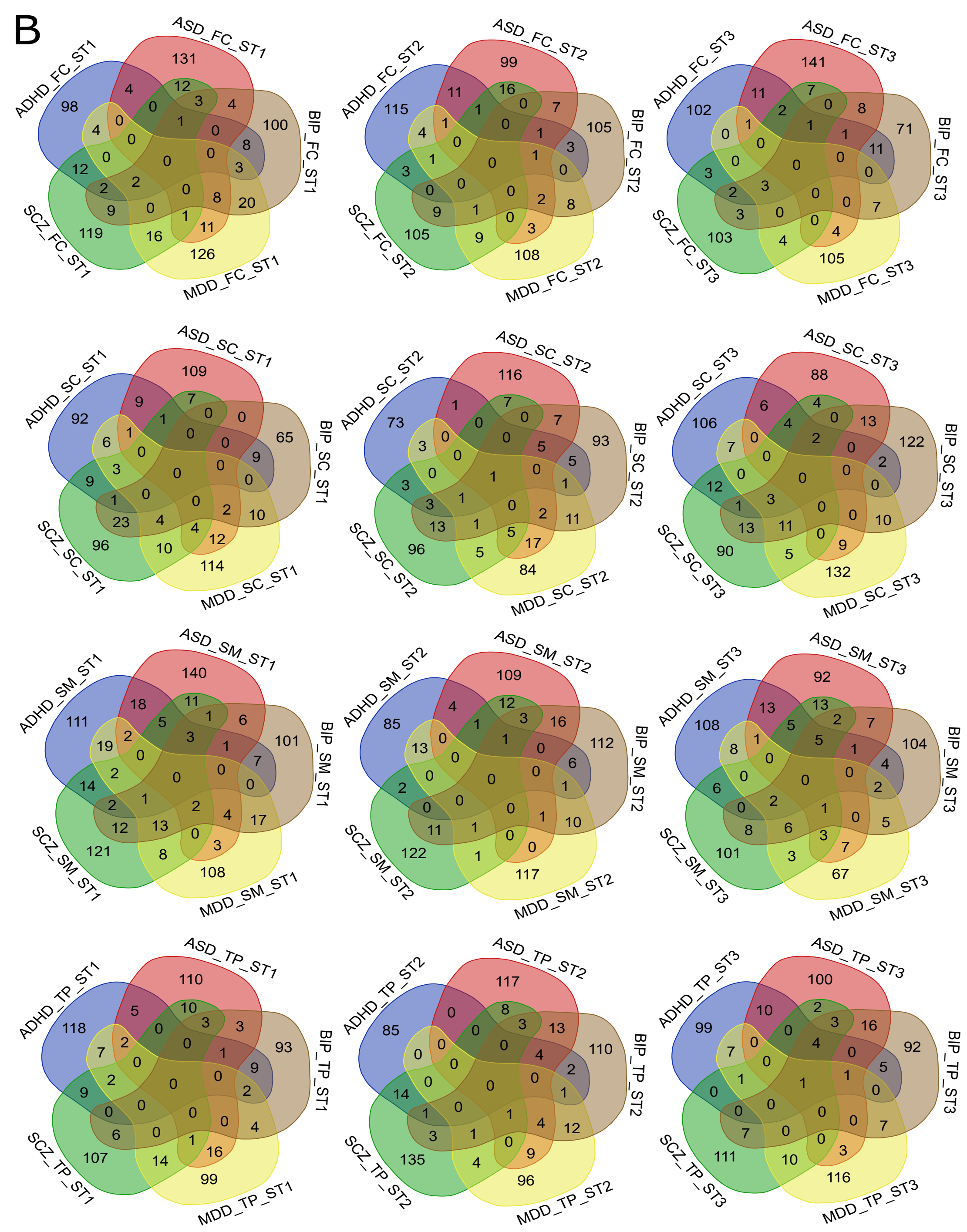

Supplement: Supplementary file 2 — Additional file 2. Venn diagram for genes from modules. (A) Venn diagram describing the overlapping modules of 12 spatiotemporal points (B) Venn diagram describing the overlapping genes from modules of 12 spatiotemporal points. Plots were generated by the online tool http://bioinformatics.psb.ugent.be/webtools/Venn/. [file 12920_2020_832_MOESM2_ESM.pdf]

A

## ADHD\_GO\_Term\_BP

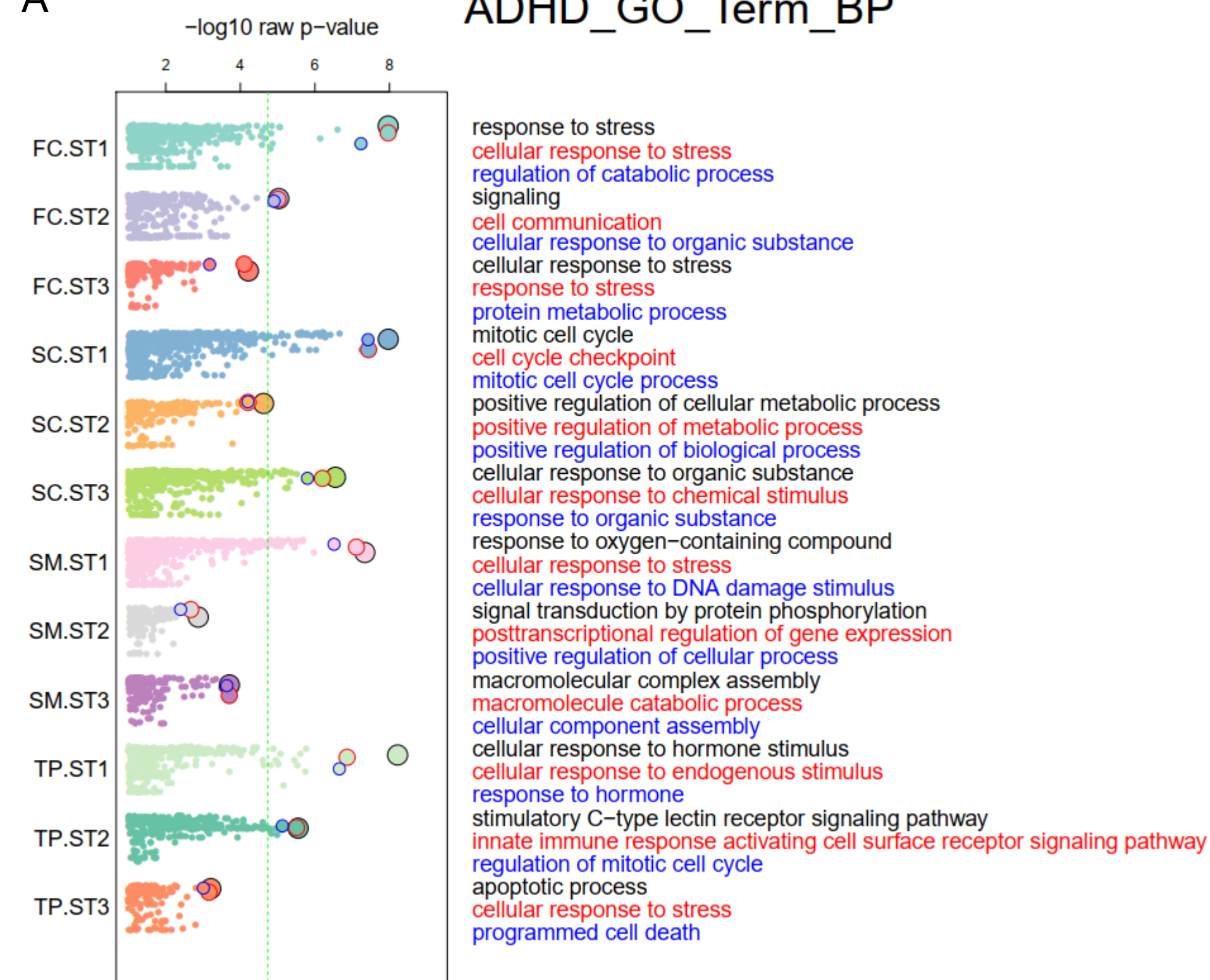

B

## ASD\_GO\_Term\_BP

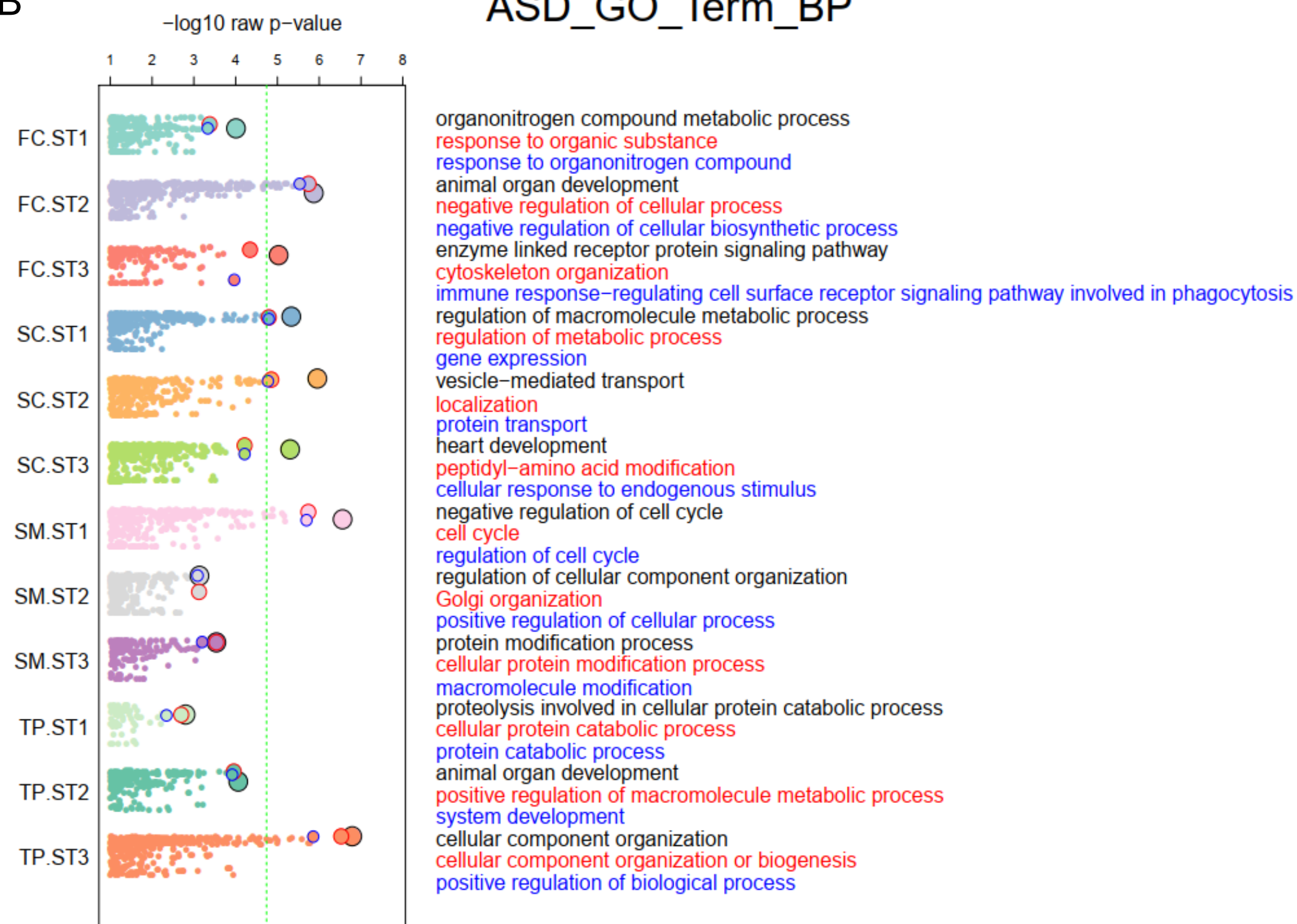

C

## BIP\_GO\_Term\_BP

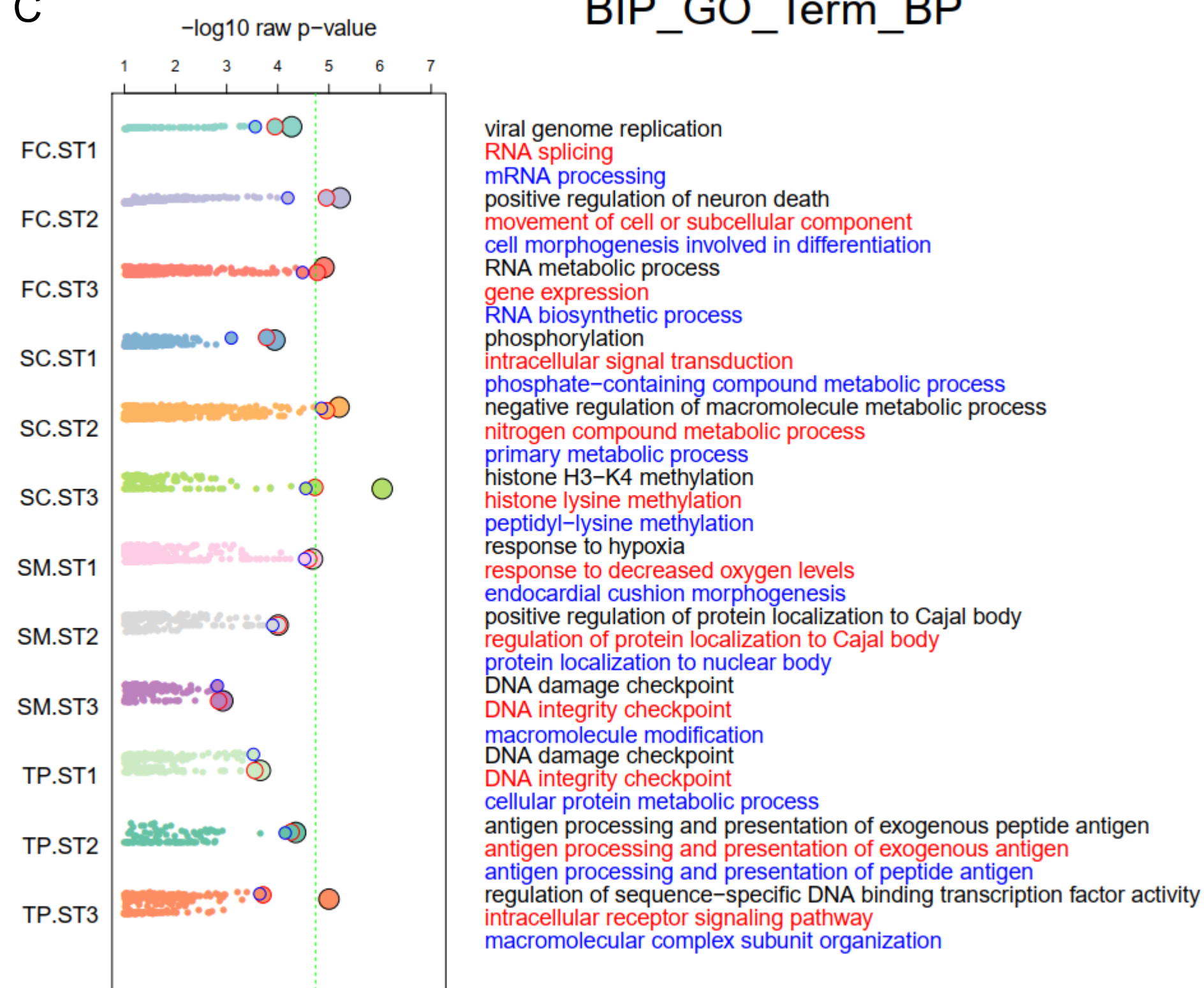

D

## MDD\_GO\_Term\_BP

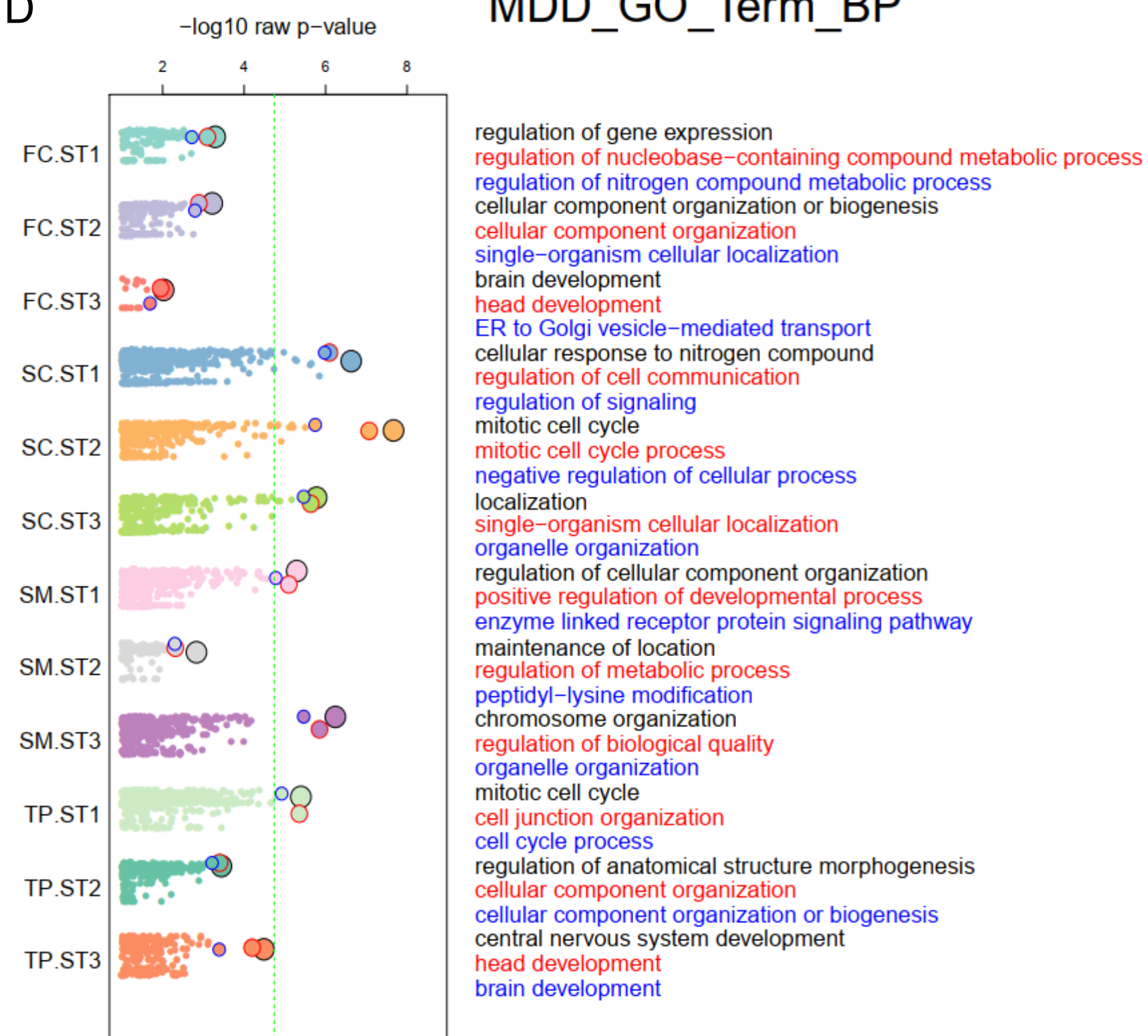

E

## SCZ\_GO\_Term\_BP

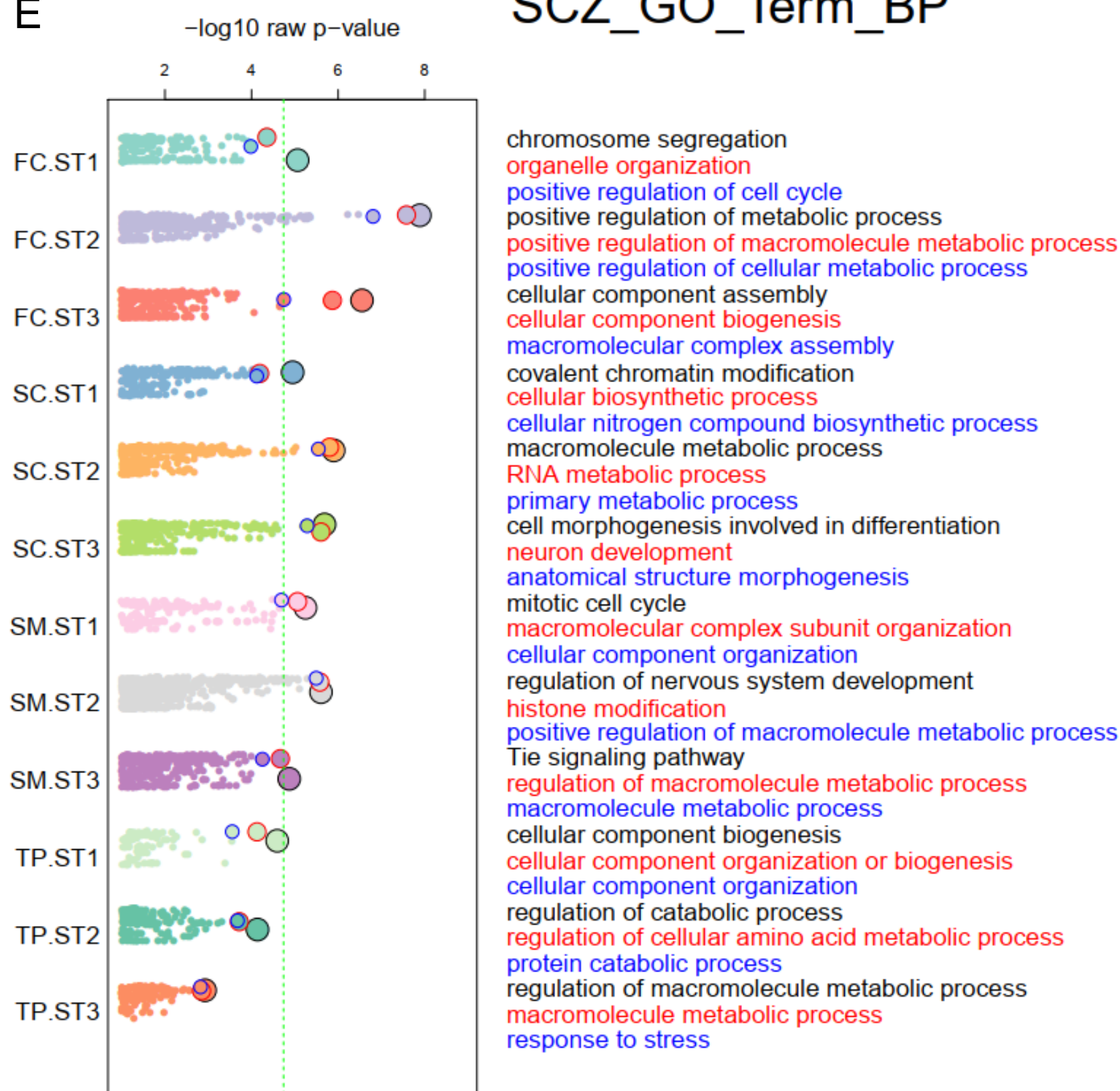

Supplement: Supplementary file 4 — Additional file 4. GO enrichment for genes (non MHC genes) in the top 10 significant modules. Gene ontology term enrichment (biological process) analysis for five mental disorders in 12 spatiotemporal points. The top three GO terms were listed on the right for each spatiotemporal point in the order of “black”, “red”, and “blue”. Green dash indicated –log10 (p-value) after Bonferroni correction of all BP terms (2740). (A) ADHD: attention deficit hyperactivity disorder; (B) ASD: autism spectrum disorder; (C) BIP: bipolar disorder; (D) MDD: major depressive disorder; (E) SCZ: schizophrenia; FC: frontal cortex region, SM: sensory motor region, SC: sub-cortical region, TP: temporal-parietal cortex region, ST1: stage 1 (prenatal), ST2: stage 2 (after birth to 11 years old), ST3: stage 3 (older than 13 years) [file 12920_2020_832_MOESM4_ESM.pdf]
